# Methodologies to Increase the Level of Evidence of Real-life Proton Therapy in Head and Neck Tumors

**DOI:** 10.14338/IJPT-20-00051.1

**Published:** 2021-06-25

**Authors:** Francesco Dionisi, Lamberto Widesott, Marco Van Vulpen, Clifton David Fuller, Rocco Frondizi, Marco Meneguzzo, Pierre Blanchard, Maurizio Amichetti, Giuseppe Sanguineti

**Affiliations:** 1 Proton Therapy Unit, Department of Oncology, Azienda Provinciale per I Servizi Sanitari (APSS), Trento, Italy; 2 Department of Radiation Oncology, Istituto di Ricovero e Cura a Carattere Scientifico (IRCCS) Regina Elena National Cancer Institute, Rome, Italy; 3 Proton Therapy Center, Delft, the Netherlands; 4 Department of Radiation Oncology, University of Texas MD Anderson Cancer Center, Houston, TX, USA; 5 Department of Management and Law, Tor Vergata University of Rome, Rome, Italy; 6 Centre for Organisational Research, Health and Public Management, Università della Svizzera Italiana (USI), Lugano, Switzerland; 7 Department of Radiation Oncology, Gustave Roussy Cancer Campus, Villejuif, France

**Keywords:** proton therapy, head and neck cancer, randomized trials, model-based approach, registries

## Abstract

This review aims to present and assess available and new methodologies to increase the clinical evidence of proton therapy data for patients with head and neck cancer. Despite the increasing number of scientific reports showing the feasibility and effectiveness of proton therapy in head and neck cancer, clinical evidence on the potential benefits of its use remains low for several reasons. In this article, the pros and cons of consolidated and new methodologies in this setting such as randomized clinical trials, the model-based approach, and the use of prospective multicentric registries will be detailed.

## Introduction

Radiation therapy (RT) has an important role in the treatment of patients with head and neck cancer, both in the definitive and the adjuvant settings [1]. In the past decades, continuous technologic advances have occurred in the field of RT with the aim of increasing precision in target volume delineation and treatment delivery and of enlarging the therapeutic window by allowing tumor dose escalation and concomitant sparing of healthy tissues [2, 3]. In this context, the use of intensity-modulated radiation therapy (IMRT) has shown in randomized clinical trials (RCTs) conducted between 2000 and 2010 to decrease acute and late toxicities for HN cancer compared with 3D conformal RT [4, 5]. For RT technologic advances, proton therapy (PT), with its unique dose-distribution profile, represents an exciting developmental field.

In the past decade, PT registered an exponential rise in both the number of patients treated and in the centers currently in operation worldwide, which exceeded 100 in July 2020 [6]. The unique properties of protons, with their finite range in tissues, along with a zero-dose beyond the end of their path, fit well for HN cancer conditions, in which the aim of RT is, in most cases, patient cure, and the risk of permanent side effects affecting a patient's quality of life (QoL) is not negligible [7]. Several national health systems allow the use of protons for specific HN subsites or in case of advanced stages of disease [8, 9], and the National Comprehensive Cancer Network HN cancer guidelines are open to the use of PT, suggesting its use when healthy tissue constraints cannot be met by photon-based RT for most subsites or, in the case of paranasal sinuses, as an alternative to IMRT [1] However, despite the increasing number of scientific reports showing the feasibility and effectiveness of the use of PT in HN cancer [10–12], the clinical evidence for its potential benefits remains low for several reasons. Radiation therapy technologies tend to progress with incremental innovations in performance and safety, in shorter development cycle than those usually needed for medical drugs, and those constant improvements in radiation technology make it difficult to evaluate it with classical approaches to generate evidence, such as clinical trials. Moreover, as underlined by the *Lancet* Oncology Commission in 2011 [13], because of the unique nature of medical device development, the use of the same criteria to evaluate pharmaceuticals and medical devices is inappropriate. Indeed, the US Food and Drug Administration requires drugs to show efficacy in a clinical trial before approval for routine use, whereas, in contrast, the most common procedure for approval of radiation oncology technologies requires only that the device vendor shows its safety for patient use.

Among professionals, the debate is intense regarding the ethical acceptability of conducting RCTs comparing protons with photons because of the superior radiation dose distribution of protons [14].

Another issue to consider when comparing cancer treatments is the cost of cancer care. The already-mentioned advances in RT technology, in addition to having increased the precision and tolerability of the treatments, have also inevitably increased the costs of cancer care. In this context, recent studies [15, 16] have shown that the direct and indirect costs of cancer care, as well as the cost of informal care, have steadily increased during the past 2 decades. Indeed, health spending on cancer has risen faster than the increase in cancer incidence, which has lead to discussions about the financial toxicity of treatments for patients, their families, and society [17]; this is even more true when it comes to a sophisticated technology, such as PT [18], in which the need for modern instruments to validate its clinical benefit and usefulness is mandatory.

In this article, we aim to present and discuss consolidated and novel approaches to adequate evidence generation for the use of PT in HN cancer: (1) randomized trials, (2) model-based methodology, and (3) registries (real-world data [RWD]).

## Randomized Trials

Randomized controlled trials represent the “gold standard” method for establishing evidence of new medical therapies. However, as mentioned in the “Introduction”, several issues exist concerning their applicability in assessing evidence of technologic advances, such as PT in comparison with conventional radiotherapy. Ethically, quoting Sullivan et al [13], “randomization of a patient to a known inferior radiation dose distribution resulting in increased irradiation of healthy tissues would be considered unethical by patients and doctors,” and this would be the case of PT versus conventional RT in HN cancer. Moreover, in recent years, small clinical studies have shown the feasibility and good initial results of PT in various challenging HN scenarios, such as oropharyngeal cancer [19], nasopharyngeal cancer [20], sinonasal, and recurrent cancer [21, 22]. The issue, of course, is more complex: supporters of RCTs claim that the clinical effect of PT's superior dose distribution remains unknown, and robust methods, such as RCTs are needed to define the clinical superiority of PT. The cost effectiveness, calculated in quality adjusted life years, offered by PT depends on several variables (related to both patients and society) and has not yet been defined [23]. Moreover, because of the few PT centers, PT cannot be offered indiscriminately to every patient with HN cancer who requires radiotherapy; in this context, RCTs could help in selecting the proper treatment (PT versus conventional RT). There are 3 types of clinical uncertainties related to the use of PT for HN cancer: (1) safety (ie, treatment toxicity for surrounding healthy tissues), (2) effectiveness (ie, tumor control), and (3) the combination of safety and treatment toxicity (ie, dose escalation, with the aim of maintaining the same toxicity level but increasing the outcome, or even dose de-escalation, with the aim of maintaining the same clinical outcome at lower toxicity levels by reducing the radiation dose). The debate regarding the proper methodology for generating clinical evidence for PT in HN cancer should be reserved for points (1) and (2). When the aim of the study is a substantial change in clinical practice, such as those studies exploring point (3), RCTs may represent the more-appropriate research strategy.

In contrast, RCTs have several well-known, intrinsic weaknesses: generalizability, costs, and duration [24]. The external validity of the results of an RCT is often a concern limiting the wider application of an RCT's findings in clinical practice [25]. Costs of RCTs are not negligible [24]: in addition, the number of PT centers is still low, limiting adequate patient enrollment in RCTs, thus compromising trial feasibility. Nevertheless, 5 RCTs [26–30] are currently ongoing evaluating the use of PT for HN cancer (Table 1). Table 1 also shows that RCT duration, especially in the context of an evolving and still scarcely available technology, such as PT, is an issue. The average estimated time to complete an RCT is more than 7 years, a period longer than the development and life cycle of a medical technology such as PT. The risk of waiting for results from RCTs that are evaluating already “old” techniques is substantial [31, 32]. For example, the trial of PT in lung cancer led by MD Anderson turned out to have negative results [33], but it might have been launched too early in the development of the technique. Indeed, when looking at the date at which the dosimetry team modified and finalized the way plans should be computed, there is a clear difference in radiation pneumonitis, favoring the more “experienced” plans. In contrast, the results of a randomized (phase IIB) trial comparing PT and RT for esophageal cancer has been published recently [34]. For the first time, the dosimetric advantage of protons was demonstrated to provide a clinical benefit in a randomized trial: a significant advantage was reported in the reduction in toxicity burden and postoperative complications in the group of patients treated with PT (80% passive-scattering technique) compared with those treated with IMRT [34].

**Table 1.** Randomized clinical trials currently open evaluating proton therapy versus conventional radiation therapy for patients with head and neck cancer.

**Study title [reference]**	**Principal institution**	**Multicenter trial**	**Clinical trial identifier**	**Estimated enrollment, No. participants**	**Study start date, y**	**Estimated completion date, y**
Photon therapy versus proton therapy in early tonsil cancer [26]	Lund University Hospital (Lund, Sweden)	Yes	NCT03829033	100	2019	2028
A phase II randomized study of proton versus photon beam radiotherapy in the treatment of unilateral head and neck cancer [27]	Memorial Sloane Kettering (New York, New York)	Yes	NCT02923570	132	2016	2021
Phase II/III randomized trial of intensity-modulated proton beam therapy (IMPT) versus intensity-modulated photon therapy (IMRT) for the treatment of oropharyngeal cancer of the head and neck [28]	MD Anderson Cancer Center (Houston, Texas)	Yes	NCT01893307	360	2013	2023
A phase III trial of intensity-modulated proton beam therapy versus intensity-modulated radiotherapy for multi-toxicity reduction in oropharyngeal cancer [29]	Institute of Cancer Research (Sutton, England UK)	Yes	ISRCTN16424014	180	2020	2028
DAHANCA 35: a national randomized trial of proton versus photon radiotherapy for the treatment of head-neck cancer [30]	Danish Head and Neck Cancer Group (Copenhagen, Denmark)	Yes	NCT04607694	108/y	2020	2025

For the HN cancer population, it is informative and helpful to look back to the history of the development of another RT technologic advance such as IMRT: a clinical effect of the dosimetric advantage (ie, the reduction of xerostomia) in parotid sparing using IMRT versus conformal RT was demonstrated on a large scale in the Parotid-Sparing Intensity Modulated versus Conventional Radiotherapy in Head and Neck Cancer (PARSPORT) multicentric randomized trial [5], which was published in 2011 after a 4-year accrual time (2003–2007). During that long period, several single-institution case series had already reported the excellent clinical results of using IMRT for patients with HN cancer [35, 36]. There is no doubt that this example could not be exactly replicated for PT clinical development in patients with HN cancer, given the major costs and the few PT centers. Nevertheless, it demonstrates that decisions about the use of new technologies should be based on the totality of available evidence and that RCTs should not be considered as the only means of generating adequate evidence. Currently, information-technology advancements (eg, the development of networks to create, analyze, and store electronic clinical records) and scientific progress (eg, the development of radiobiologic models to compare PT and RT) contribute to offer new methodologies helpful for generating evidence from PT clinical data, which will be presented in the following paragraphs on the model-based approach and the development of registries.

## The Model-Based Methodology

Other methodologies, apart from RCTs, should be applied when evaluating the development of new medical devices. In the context of RT, in silico planning comparative studies have been extensively used to analyze and report the different dose distribution of investigated techniques [37–39]. The relationship between dose distribution and the development of radiation-induced side effects is usually described, when available, by normal tissue complication probability (NTCP) models [40]. Regarding HN radiotherapy, NTCP models have been developed for the estimated risk of major RT-related side effects that have an effect on the QoL of patients, such as xerostomia, dysphagia, and tube feeding dependence [41–43]. In 2013, the Dutch group, Langendijk et al [44] first proposed a stepwise, model-based methodology for selecting patients for PT. Essentially, the 3 NTCP models cited here were selected and used for the estimation of toxicity reduction between the best-of-the-art IMRT plans and PT plans. Changes in NTCP values (ΔNTCP) that justify the use of PT were set ≥ 10% for grade ≥ II and/or ≥ 5%, for grade ≥ III side effects, respectively. Considering the above-mentioned issues in conducting RCTs in PT, this approach represents a valid methodology to tailor the indications for PT based on validated models. An external validation of photon-derived NTCP models in a HN PT cohort was evaluated at MD Anderson in 2016 [45], demonstrating the validity of a model-based approach for the selection of patients with HN for PT. In this context, considering the costs of protons, the model-based approach could refine selection for PT, thus limiting the use of such an expensive and still-rare technology to only when a clinical benefit is expected. It could also serve for patient selection to be included in an RCT, as in the DAHANCA 35 trial (Table 1) [30]. Patients selected for PT with this approach must be followed in prospective studies and registries (see “Registries”) for clinical validation of any benefits. Moreover, a methodology based on models requires continuous data collection, which ensures adequate tailoring of the NTCP models to fit with differences in patient selection, concomitant treatments, and radiation techniques adopted over time. Of note, the first report about the clinical implementation of this methodology was published recently in August 2020 [46]. Tambas et al [46] reported the experience of the Dutch group with the model-based approach for selecting patients with HN for PT: the methodology was judged feasible, although time consuming, especially during the learning-curve period. Patients qualified for PT had more locally advanced disease, usually pharyngeal tumors. With this approach, 35% of patients with HN were referred to PT based on a ΔNTCP value higher than the threshold, mostly for dysphagia and xerostomia.

### Registries

As mentioned, because of the continuous technologic improvements and for ethical reasons, a “traditional” clinical trial may be impractical or excessively challenging to conduct for radiotherapy technologies and, in particular, for PT. As proposed by US Food and Drug Administration guidance [47], analyses of RWD with appropriate methods can sometimes provide similar information with comparable or even superior characteristics to information collected and analyzed through a traditional clinical trial. The RWD sources can be registries, collections of electronic health records, and administrative and health care claims databases. However, not all RWD are collected and maintained in a way that provides sufficient reliability.

Observational studies, such as registries, have several advantages over RCTs, such as costs, generalizability, long-term outcomes, and relevant outcomes, rather than intermediate outcomes, among others. In particular, long-term follow-up of effectiveness and safety outcomes is relevant when RCTs (generally focused on short-term outcomes) or model-based approaches (mainly focused on toxicities) are combined with registries. Moreover, as demonstrated by Concato et al [48], although the hierarchy of study designs considers the results of observational studies to be of lower quality than that of RCTs, well-designed observational studies are capable of reproducing the results of RCTs without overestimating the magnitude of the effect of the treatment.

However, registries and model-based approaches are prone to selection bias because numerous factors may influence the enrolment of patients in a registry and may be difficult to identify in advance and to prevent.

Registries should contain all the information relating to the technology used account for its evolution, costs, and clinical outcomes. As underscored by Porter et al [49], there is no consensus on which outcomes are most important; consideration of outcomes that matter to patients, aside from survival, remains limited. A minimum sufficient set of outcomes for patients with HN cancer, with well-defined methods for their collection and risk adjustment, is needed, and then, that set needs to be standardized globally within the radiotherapy community. Table 2 reports on a proposal for minimum data elements to be included in registries for PT for patients with HN cancer: RWD should include patients and tumors clinical characteristics as well as clinical outcomes and patient-reported outcomes. Moreover, some technical aspects should be included in the register to appropriately characterize the PT plan delivered to the patients (eg, passive scattering versus active scanning use, single-field-optimized versus multifield-optimized plans, robustly optimized versus planning target volume (PTV)-based plans, inclusions of the margins implemented for PTV or robust optimization, among others). In addition, dose-volume histograms and dose distributions in Digital Imaging and Communications in Medicine format should be provided for subsequent independent analyses. Adequate patient-protection procedures should be put in place and strictly followed to preserve patient privacy.

**Table 2.** Proposal of minimum data elements for head and neck (HN) proton therapy (PT) registries.

**Data element**	**Detail**
Population characteristics	
Sex/age	
Performance status	ECOG
Comorbidities	List + Charlson CMI
QoL	EORTC QLQ-C30/EORTC QLQ-HN35, other
Tumor characteristics	
HN site	
Diagnosis	ICD-10 [50]
Staging	TNM, 8th edition [51]
Oncologic treatments (apart from PT, check all that apply)	
Surgery	Specify (intent, type, date)
Chemotherapy	Specify (intent, type, dose, duration)
Immunotherapy	Specify (intent, type, dose, duration)
PT indication (check all that apply)	
Multidisciplinary tumor board	
Photon-proton dosimetric comparison in favor of PT	Provide DICOM file
Model-based approach	
Patient under clinical trial	Specify
Other	Specify
PT intent (check all that apply)	
Definitive	
Postoperative	
Palliative	
Proton treatment technical characteristics (check all that apply)	
Proton passive scattering	
Proton active scanning: multifield or single-field optimization	
PTV-based optimization: PTV margins, mm	Specify
Robust optimization: shifts, mm, and range, %, mm	
Daily imaging	Specify (3D, 2D, automatic, manual, etc)
Fixation devices	Specify
Proton fields, No.	
Dose recalculation on 3D imaging, No.	
Replanning, No.	
Fractions planned, No.	
Fractions delivered, No.	
Start date of treatment	MMDDYYYY
End date of treatment	MMDDYYYY
Anatomic site of each prescribed dose level	
Total dose planned for each prescribed dose level	
Total dose delivered for each prescribed dose level	
DVH and dose distribution	DICOM file
PT outcome	
Patient status	NED, alive with disease, dead from cancer, dead from other causes (specify)
Local control	y/n
Disease-free survival	y/n
FUP duration	
Toxicity	List, CTCAE, version 5.0
Performance status	ECOG
QoL	EORTC QLQ-C30/EORTC QLQ-HN35, other
Costs	
* *Construction costs (project management, equipment, building, infrastructure)	Quantify
* *Operation costs (personnel fixed and variable costs, equipment and building operation, building costs, other)	Quantify

**Abbreviations:** ECOG, Eastern Cooperative Oncology group; CMI, Comorbidity Index; QoL, quality of life; EORTC, European Organisation for Research and Treatment of Cancer; QLQ, quality of life questionnaire; ICD, International Classification of Diseases; DICOM, Digital Imaging and Communications in Medicine; PTV, planning target volume; DVH, dose-volume histogram; NED, no evidence of disease; y/n, yes/no; FUP, follow-up period; CTCAE, Common Terminology Criteria for Adverse Events (National Cancer Institute, Bethesda, MD).

To ensure the reliability of the RWD, registries should have relevant time windows for data element collection (ie, a common temporal framework). An aid in the search for standardization of outcome measures could come from the International Consortium for Health Outcomes Measurement (ICHOM) [52] and the American Society for Radiation Oncology (ASTRO) [53] experiences: their role is not to devise new outcomes measures but to agree on which measures are well validated, including patient-reported measures (ie, outcomes of interest at the patient level), which all studies should use. To date, ICHOM has defined 39 standard sets for many pathologies (ranging from atrial fibrillation to COVID-19); unfortunately, they have not yet defined a standard set for patients with HN cancer. The ASTRO document aims to define the most-relevant RT data elements to be entered in the oncology information system and exchanged among electronic systems. Table 2 combines several global sets of outcome measures that matter most to patient (eg, survival, recurrence-free survival, toxicity, performance status, and European Organisation for Research and Treatment of Cancer [EORTC] QoL questionnaires) with the ASTRO recommendations, expanding them, contextualizing the recommendations to RT and PT with external beams (ie, no data relating to brachytherapy is required). Newer, interesting efforts are represented by the European Society for Radiation Oncology and European Organisation for Research and Treatment of Cancer E2-RADIatE platform, a pan-European standardized radiation oncology data infrastructure that is currently being developed for patients with oligometastatic disease [54] and those undergoing PT (ParticleCare project). The E2-RADIatE project aims to establish a comprehensive system of oligometastatic disease-characterization factors that should be assessed in all patients treated with radical local treatment for oligometastatic disease. The E2-RADIatE platform is an interesting example of consensus among various scientific societies. However, it refers only to the clinical characterization and classification of oligometastatic disease, and no description of the treatment, clinical outcome, or cost effectiveness among other outcomes is proposed.

One of the main issues with observational data is that it is difficult to reliably compare 2 treatments because of “confounding by indication,” meaning patients receive a certain treatment (eg, protons versus photons) for a reason. Therefore, more-sick individuals may receive protons, whereas healthier individuals receive photons. If so, a direct comparison could show a benefit for photons, but that effect could, in part, be attributed those being treated with photons being healthier. Therefore, confounding by indications needs to be assessed. Obviously, the best way to do that is by randomization, as explained in the “Randomized Trials.” The model-based approach also accounts for this difficulty by comparing between expected toxicity and observed toxicity in the same patient. Regarding registries, the issue can (partially) be corrected in the statistical analyses.

To minimize bias, all the data included in the registries “should be accurate, as complete as possible, and have an appropriate scope to address the question at hand (i.e., data adequacy)” [47]. Being such an expensive technology, PT data collection should include all relevant health-economy parameters [55] useful for conducting cost-effectiveness analyses.

For a proper analysis of RWD, some tools are essential, such as (1) a predefined common set of data elements, (2) a common definitional framework for common understanding of information, and (3) the choice of predefined time intervals for data collection and research analyses. We have 2 conflicting needs: registries cannot be too general or synthetic because of the risk of being uninformative and incurring bias; however, they must not be too complex and detailed to be introduced into clinical practice and in different health contexts. It is important not only to identify a new working data set but also to plan for integration and use in current systems.

## Conclusions

The use of PT for patients with HN cancer could improve the QoL of survivors by reducing treatment toxicity. The process of increasing the level of evidence for its use is complex because (1) being a technologic advancement, the use of RCTs could be limited and prone to ethical and practical issues as described; (2) because of the high costs of PT, a high level of evidence is required by national health systems and insurance companies before investing in its full implementation; and (3) although increasing, the number of patients with HN treated by any single PT center is low, thus compromising the possibility of evidence generation by traditional, hierarchic methodologies.

As described previously, every solution proposed has pros and cons and is prone to criticisms and risks of bias (Table 3). Integration among all methodologies probably represents the best strategy for optimizing the use of clinical data of patients with HN cancer treated with PT to increase the quality of the evidence. Among the 3 methodologies analyzed, the model-based approach is the only one that has already been fully implemented for patients with HN cancer. A recent publication has demonstrated the clinical feasibility of this combination approach in selection of patients with HN cancer for PT. The need for continuous refinement of the model represents, at the same time, a time-consuming complication for the broad development of this approach and an opportunity to obtain a scientific and rigorous approach for patient selection, which could assist in the development of more-tailored RCTs or even replace them when the model approach is combined with rigorous prospective data collection and examination. In the context of clinical data collection and reuse for clinical-evidence generation, it is of interest to introduce the Rapid Learning concept, which involves the reusing of clinical data to develop knowledge in the form of models that can predict treatment outcomes with the use of machine-learning technologies [56]. Regarding traditional RCTs, given the difficulty of running RCTs for technology evaluation, it appears essential that groups collaborate to allow mutualization of data through prospective meta-analyses. It is unlikely that one trial alone will set a new standard of care, but rather, the combination of trials is needed, as used in the past with HN cancers for chemotherapy [57] or radiotherapy fractionation [58]. The strong internal validity of RCTs and their potential combination through meta-analysis will also allow to “data mining” and generation of hypotheses based on rigorously collected data. There is still a need for well-conducted, fair multicentric RCTs with clear objectives and limited durations: the increasing number of PT centers will likely expedite their implementation. All clinical and technical data (including costs) of patients treated with PT should be collected in prospective, well-designed, ad hoc–generated, multisource registries, with essential periodic analysis to facilitate the understanding of clinical evidence and effectiveness (including cost effectiveness) of PT for patients with HN cancer.

**Table 3:** Pros and cons of proposed methodologies to assess evidence for proton therapy in patients with head and neck cancer.

**Methodology**	**Pros**	**Cons**
RCTs	Consolidate “gold standard” to generate evidence	Lack of generalizability, costs, ethics, duration, short-term outcomes, feasibility and appropriateness in technology assessment
Model-based approach	Practical and feasible approach	Uncertainties related to the model, time consuming, risks of bias, possibility of NTCP model variation over time
Registries	Comprehensive, real-world data (clinical, technical, costs); more-generalizable, long-term outcomes	Need to establish common ontology, time consuming, risks of bias

**Abbreviations:** RCT, randomized clinical trial; NTCP, normal tissue complication probability.
